# Lipid Metabolism in Plants

**DOI:** 10.3390/plants9070871

**Published:** 2020-07-09

**Authors:** Hyun Uk Kim

**Affiliations:** Department of Bioindustry and Bioresource Engineering, Plant Engineering Research Institute, Sejong University, Seoul 05006, Korea; hukim64@sejong.ac.kr; Tel.: +82-2-6935-2491

**Keywords:** lipid, fatty acid, triacylglycerol, glycerolipid, acyltransferase, transcription factor

## Abstract

In plants, lipids function in a variety of ways. Lipids are a major component of biological membranes and are used as a compact energy source for seed germination. Fatty acids, the major lipids in plants, are synthesized in plastid and assembled by glycerolipids or triacylglycerols in endoplasmic reticulum. The metabolism of fatty acids and triacylglycerols is well studied in most Arabidopsis model plants by forward and reverse genetics methods. However, research on the diverse functions of lipids in plants, including various crops, has yet to be completed. The papers of this Special Issue cover the core of the field of plant lipid research on the role of galactolipids in the chloroplast biogenesis from etioplasts and the role of acyltransferases and transcription factors involved in fatty acid and triacylglycerol synthesis. This information will contribute to the expansion of plant lipid research.

## 1. Introduction

Plant lipids are diverse and essential for cells. They are essential for the integrity of cells and organelles by acting as a hydrophobic barrier for the membrane. In addition, lipids are stored in the form of chemical energy in seeds. Furthermore, they act as a signal molecule to regulate cell metabolism [1,2]. The main form of lipid in plants is the glycerolipid in which the carboxyl group of the fatty acid is ester-linked with the hydroxyl group of glycerol. Lipid synthesis involves several organelles in a cell. Fatty acid is synthesized from chloroplasts, and is directly combined with glycerol to become a galactolipid, a major component of the chloroplast membrane, and fatty acids are transferred to the cytoplasm to bind with glycerol in the endoplasmic reticulum (ER) to become a phospholipid of the cell membrane [3]. Between the ER membranes of the seed cells, triacylglycerol (TAG) is synthesized and stored in the oil body [4]. In addition, in the ER of the epidermal cell, fatty acid is transformed into components of cutin and wax, which are lipids of the cuticle layer that prevent water loss [5]. Fatty acid, glycerolipid, and triacylglycerol biosynthesis pathways have been identified through genetic analysis using Arabidopsis mutants [6]. Recently, researchers have been conducting research on the discovery of transcription factors that regulate lipid synthesis [7], the role of galactolipids in photosynthesis [8], cuticle lipid synthesis and transport [9,10], and lipid remodeling during development and stress [11]. To improve the quality and quantitative traits of plant oil in terms of application, studies on the modification of fatty acid composition and the enhancement of oil content are underway [12,13].

This Special Issue introduces the following: the role of galactolipids during the conversion from etioplast to chloroplast under light conditions; Phospholipase A and its association with lignin metabolism; the roles of GPAT9 and PDAT1 involved in TAG synthesis; an integrated review of WRINKELD1 (WRI1), a master transcription factor that regulates TAG synthesis; and a study on TAG synthesis under stress regulation by the MYB96 transcription factor.

## 2. Highlight of Special Issues

Fujii et al. [14] discussed the role of galactolipids, monogalactosyldiacylglycerol (MGDG), and digalactosyldiacylglycerol (DGDG), in membrane formation in the transition of plastid differentiation from dark to light condition. The process of transforming etioplasts of dark-grown cotyledon to chloroplasts after light expose to the mutants, whose synthesis of galactolipids was inhibited, was observed. It is suggested that galactolipids play a crucial role in the accumulation of chlorophyll biosynthesis and light-harvesting proteins, as etioplasts receive light and transform the internal membranes of prolamellar bodies (PLBs) and prothylakoids (PTs) into thylakoid membranes. This review is an advance that explains why galactolipids exist in photosynthetic plants predominantly present in plastids.

Jang and Lee [15] revealed that Arabidopsis patatin-related PLAIIIα affects the lignification of Arabidopsis and polar xylem. Phospholipase A (PLA) consists of two groups in plants. Low-molecular-weight PLA_2S_ and patatin-related PLA (pPLA) hydrolyze the acyl ester bonds of the glycerolipids, releasing free fatty acids and lysophospholipids [16,17]. The pPLAs participate in a number of cellular processes, such as signal transduction, cell growth regulation, membrane remodeling in response to environmental stress, and lipid metabolism by hydrolyzing membrane glycerolipids [17,18,19]. In this study, the lignin content was reduced and the ethylene biosynthesis synthetic gene expression was upregulated in the overexpressed *PLAIIIα* in Arabidopsis transgenic plants. The overexpression of *PLAIIIα* in the woody plant poplar downregulated *MYB92* and *MYB152*, the regulators of lignin synthesis. These results suggest that the hydrolysis products of glycerolipids by PLA regulate lignin synthesis. In the future, the discovery of signaling molecules induced by PLA that regulate lignin synthesis is expected.

Yang et al. [20] studied the metabolism of membrane lipid synthesis of *Physcomitrella patens*, a moss corresponding to early-stage plants during the terrestrial evolution of plants. The haploid cells of *P. patens*, protonema, and gametophore, have a high content of 65% of the long-chain polyunsaturated fatty acid (LC-PUFA) and a very-long-chain-PUFA [21,22,23]. *P. patents* GPAT9 specifically delivers PUFA from the PUFA-CoA pool, to the hydroxyl group at the *sn-1* position of glycerol-3-phosphate during glycerolipid synthesis. The *PpGPAT9* was introduced into Arabidopsis to further increase the seed oil content by 10% and further increase the PUFA composition of seed oil by 60%. This suggested that PpGPAT9 can be used to increase the oil content of oilseed seeds through genetic engineering.

Fan et al. [24] studied carbon flux between lipids and sugar. The mutation of ADP-glucose pyrophosphorylase1 (ADG1) does not form a starch. The *adg1* mutant does not use starch as an energy source, and thus shows growth inhibition under day/night conditions [25,26]. TAG was synthesized in vegetative tissue by overexpressing phospholipid:diacylglycerol acyltransferase1 (PDAT1) [27] in the *adg1* mutant. The fatty acid produced by TAG decomposition in dark conditions was used as an energy source for cellular respiration, thereby reducing the growth inhibition of the *adg1* mutant under day/night conditions. In the future, in-depth research on carbon remobilization between TAG and starch is expected.

Lee et al. [28] suggested that the MYB96 transcription factor not only regulates the oil content during seed development [7], but also controls the synthesis of TAG in drought stress in vegetative tissue leaves. TAG accumulation was very high in Arabidopsis seedlings where MYB96 was constitutively overexpressed. *MYB96* is induced by ABA to directly regulate *PDAT1* and indirectly regulate *diacylglycerol acyltransferase1* (*DGAT1*), the last two enzyme genes of TAG synthesis, to induce TAG synthesis in leaves. TAG accumulation in vegetative tissue under stress will contribute to the optimization of energy metabolism and the sequestering of toxic fatty acids degraded by membrane lipids. Research on the metabolism of TAG under stresses is a research area that has recently been focused on.

Kong et al. [29] reviewed the integrated regulatory mechanism of WRINKLED1, the primary transcriptional regulator of plant oil biosynthesis. In 1998, Benning’s lab first reported that the *wri1-1* mutant is a transcription factor that regulates seed oil content due to finding that the oil content is reduced by 80% compared to wild type seeds [30]. Subsequently, it was found that WRI1 regulates many genes involved in glycolysis and fatty acid biosynthesis at the level of transcription regulation [31]. Recently, it was found that WRI1 regulates target gene expression through interaction with Mediator Subunit 15 (MED15) [32] and BTB/POZMATH (BPM) [33] regulators, or through phosphorylation by kinase KIN10 [34]. WRI has been studied as a candidate transcription factor that can be used in the development of biotech crops to improve oil production [35]. The creation of the WRI1 new allele with enhanced activity through targeted base editing in the regulatory domain of WRI1 suggests the possibility for crop development with enhanced oil content.
